# Routine pathologic evaluation of circular stapler anastomotic rings is not useful after resection for colorectal cancer: retrospective study and systematic review with meta-analysis

**DOI:** 10.1093/bjsopen/zrac122

**Published:** 2022-10-12

**Authors:** James R Holden, Pam McIntosh, Garrett G R J Johnson, Jason Park, David J Hochman, Ashley Vergis, Benson Yip, Ramzi M Helewa, Eric Hyun

**Affiliations:** Department of Surgery, University of Manitoba and St. Boniface Hospital, Winnipeg, Manitoba, Canada; Department of Surgery, University of Manitoba and St. Boniface Hospital, Winnipeg, Manitoba, Canada; Department of Surgery, University of Manitoba and St. Boniface Hospital, Winnipeg, Manitoba, Canada; Clinician Investigator Program, University of Manitoba, Winnipeg, Manitoba, Canada; Department of Surgery, University of British Columbia, Vancouver, British Columbia, Canada; Department of Surgery, University of Manitoba and St. Boniface Hospital, Winnipeg, Manitoba, Canada; Department of Surgery, University of Manitoba and St. Boniface Hospital, Winnipeg, Manitoba, Canada; Department of Surgery, University of Manitoba and St. Boniface Hospital, Winnipeg, Manitoba, Canada; Department of Surgery, University of Manitoba and St. Boniface Hospital, Winnipeg, Manitoba, Canada; Department of Surgery, University of Manitoba and St. Boniface Hospital, Winnipeg, Manitoba, Canada

## Abstract

**Background:**

Circular staplers are commonly used for reconstruction after radical resection for colorectal cancer. Pathological analysis of the anastomotic rings is common practice, although the benefits are unclear. The purpose of this study was to evaluate the usefulness of routine histopathological analysis of anastomotic rings in an original series and in a systematic review of the literature.

**Method:**

The retrospective study was performed at two university-associated academic hospitals in Winnipeg, Canada, including patients investigated for colorectal cancers (within 30 cm of the anal verge) who underwent resection between 2007 and 2020. The systematic review involved Ovid MEDLINE, Embase, Scopus, and Web of Science databases, selecting for adult human studies involving analysis of anastomotic rings in elective colorectal cancer resections. The main outcome measure was the proportion of patients with cancer in the anastomotic ring specimens. The frequency of benign pathology findings and changes to patient management were also examined.

**Results:**

Out of 673 eligible patients, 487 were included in the retrospective analysis. No patients had cancer within the anastomotic ring specimens. Twenty-five patients (5.1 per cent) had benign pathological findings within the anastomotic ring specimens, and patient management was never affected. In the systematic review, 27 articles were included in the final analysis out of 5848 records reviewed. The rate of cancer within anastomotic ring specimens was 0.34 per cent, and the rate of change in patient management was 0.19 per cent.

**Conclusion:**

The likelihood of finding cancer within anastomotic rings is rare and their histopathological examination seldom changes patient management.

## Teaser

This study was designed to evaluate the usefulness of sending circular stapler anastomotic rings for histopathological analysis. It included a retrospective study of 487 patients and a systematic review of the literature. Our results indicate that finding cancer within the anastomotic ring specimens is rare, and alterations to patient management because of these results is even rarer.

## Introduction

End-to-end anastomosis (EEA) staplers are commonly used to restore intestinal continuity after resection for left-sided colorectal cancers^1^. Firing of the EEA stapler creates two rings of tissue, often termed ‘donuts’ or ‘doughnuts.’ The proximal ring originates from the distalmost end of the bowel proximal to the specimen, whereas the distal ring originates from wherever on the rectal or colorectal stump the ‘spike’ of the stapler is punctured through. Intraoperative assessment of these rings by surgeons predicts an intact anastomosis^1^. Additionally, many surgeons routinely send these anastomotic rings for histological analysis to provide supplementary pathological information to that of the main surgical specimen, although this practice incurs a cost and requires considerable time and effort on the part of a pathologist^2,3^.

Previously, multiple studies in the literature have investigated the value of this examination, with results generally showing little utility to analysing EEA stapler anastomotic rings^2–13^. Nonetheless, guidelines from the UK’s Royal College of Pathologists continue to recommend routine analysis of these tissue samples, and surgical guidelines on the subject are lacking^14^. The purpose of this study was to retrospectively examine data at two institutions regarding the utility of histopathological analysis for anastomotic rings, and to comprehensively review the available literature on this practice. It was hypothesized that neither local nor systematic review data would support routine histopathological analysis of anastomotic rings.

## Methods

### Retrospective analysis

#### Study protocol

The retrospective chart review was approved by the University of Manitoba’s Health Research Ethics Board and St. Boniface Hospital’s Research Review Committee. The STROBE tool was used as a reporting guide^15^.

#### Study participants

All patients who underwent an elective anterior resection or low anterior resection (LAR) for colorectal cancer using a circular stapler anastomosis from February 2007 to December 2020 at St. Boniface Hospital and Victoria Hospital in Winnipeg, Manitoba, Canada were included. Both institutions are university-affiliated teaching centres with a high volume of patients referred for subspecialized surgical care. Patients were identified retrospectively based on billing codes of the hospitals’ colorectal surgery group, then reviewed for inclusion criteria. Data were collected through manual review of hospital electronic medical records and surgeons’ office charts. The timeframe was selected because it marked the onset of a significant volume of colorectal procedures using the EEA stapler at these institutions. Tumour location was reported based on preoperative MRI imaging or, if unavailable, preoperative endoscopy reports. Tumour location was defined based on distance from the anal verge, similar to previous studies^13^. The following were selected as exclusion criteria: tumour location less than 30 cm from the anal verge (to capture all cases where the EEA stapler would conceivably be used), benign disease, non-sphincter-preserving surgery, handsewn anastomosis, anastomotic rings discarded intraoperatively, and absent histological examination report for anastomotic rings. Anastomotic ring evaluation always occurred after fixation in a formalin solution. The following variables were collected for each patient: age, sex, height, weight, BMI, ASA grade, tumour stage, tumour heigh from anal verge, tumour size, tumour histology, tumour differentiation, presence of neoadjuvant treatment, surgical approach, intraoperative leak test results, anastomotic reconstruction method, surgical margins, and total mesorectal excision (TME) completeness. The TME completeness was evaluated by a trained pathologist in accordance with the methods described by Quirke *et al.*^14^.

### Outcomes

The primary outcome was the presence of cancer within the anastomotic ring specimens. Secondary outcomes included benign abnormalities within the anastomotic ring specimens and any changes in patient management specifically arising from the results of the anastomotic ring pathology. Pathology outcomes were evaluated based on the reporting of anastomotic ring specimens from institutional pathologists. All specimens were examined in accordance with best practices of the Canadian Association of Pathologists, using transverse sectioning of anastomotic ring specimens at regular intervals. Cancer staging was performed using the American Joint Committee on Cancer system based on the final surgical pathology. Patient medical records were examined to determine whether any change in clinical management (any additional surgery, chemotherapy, radiation, or other documented treatment modification) occurred based on anastomotic ring histopathology.

In 2020, the pathology department associated with the participating hospitals adopted a policy of reporting gross pathology results only for anastomotic rings where the tumour is less than 2 cm from the main specimen margin, a change from the previous practice of routine microscopic examination of all anastomotic rings. Patients who had only microscopic pathology results reported for their distal anastomotic ring were included in the study.

### Systematic review

#### Study protocol and design

The systematic review protocol was registered in the international prospective register of systematic reviews (PROSPERO 2021, CRD42021275722). The systematic review was conducted according to guidelines enumerated in the Methodological Expectations of Cochrane Intervention Reviews and reported according to the PRISMA guidelines^16,17^. The research questions developed *a priori* were as follows. In adult patients undergoing elective colorectal resection for cancer, how often is cancer found in the anastomotic ring specimens?; how often is any other pathological abnormality found in the anastomotic ring specimens?; and how often does pathological analysis of anastomotic ring specimens alter patient management?

This research included all studies with adult patients (18 years or older) who underwent elective colorectal surgery for colorectal adenocarcinoma. For cohorts where some patients met the inclusion criteria and others did not (for example mix of children and adults), those where 80 per cent or more met the inclusion criteria were included in the analysis. Patients had to have undergone elective resection of their left colon or rectal cancer with curative intent, had their anastomosis constructed using a circular stapler, and had the tissue rings from the stapler sent for histopathological analysis. All practice settings were included. Patients who did not have sphincter-preserving surgery, had a handsewn reconstruction, or did not have anastomotic rings sent for pathological analysis were excluded. Trials of emergent operations and animal trials were also excluded.

A search strategy was designed in consultation with an independent health sciences information professional (*Fig. S1*). Database searches were conducted in MEDLINE (Ovid), Embase (Ovid), Cochrane Central (Ovid), Scopus, and Web of Science. A search of the grey literature was conducted, including American Society of Colon and Rectal Surgeons conference proceedings and unpublished/ongoing clinical trials identified from ClinicalTrials.gov. Literature was searched from 1975 (the year that a circular stapler was first described in the literature) until 23 September 2021^18^. All retrieved records were imported into EndNote (X9, Thomson Reuters, Carlsbad, CA, USA) and deduplicated.

Citations were imported into Covidence (Covidence.org). Two reviewers independently screened citations for eligibility in duplicate using a two-stage approach. First, titles and abstracts were reviewed. Potentially relevant full-text articles were examined to determine whether they met inclusion criteria. The rationale for full-text article ineligibility was recorded. Conference abstracts were eligible for inclusion if the primary outcome was available from presented data and no full-text article based upon the data had been published to date. Data were extracted by two independent reviewers using a standardized pilot-tested form. Disagreements at all phases were resolved through consensus, or with assistance from a third party if consensus could not be achieved.

#### Outcomes

The primary outcome was the proportion of anastomotic rings where cancer was detected. Secondary outcomes were other pathological findings in the anastomotic rings and changes to patient management based on anastomotic ring pathology (same comment as above). Studies were excluded if outcomes were unavailable from the full-length report or study abstract.

#### Risk of bias

Bias assessment was conducted by two independent reviewers using a modified Newcastle–Ottawa scale for assessing the quality of non-randomized studies in meta-analyses (*Table S1*)^19^. Discrepancies between reviewers were resolved by discussion and, if necessary, a third investigator (EH). Risk of bias was assessed with respect to the primary outcome of this systematic review only.

#### Statistics

Patient demographic information and outcomes of interest were analysed using basic descriptive statistics. All continuous data were normally distributed and are reported using mean and s.d. Categorical variables are reported using sample proportions. The analysis plan was determined *a priori.* A Freeman–Tukey transformation was used to calculate the weighted summary proportion of positive anastomotic rings, and changes to patient management^20^. Meta-analysis data were subjected to calculations using both fixed and random-effects models and are reported as random-effects models in the text. Statistical heterogeneity was explored and quantified using the *I*^2^ test^21^. Data were analysed using MedCalc version 20.026 (MedCalc Software, Ostend, Belgium).

## Results

### Retrospective study

**Fig. 1:**
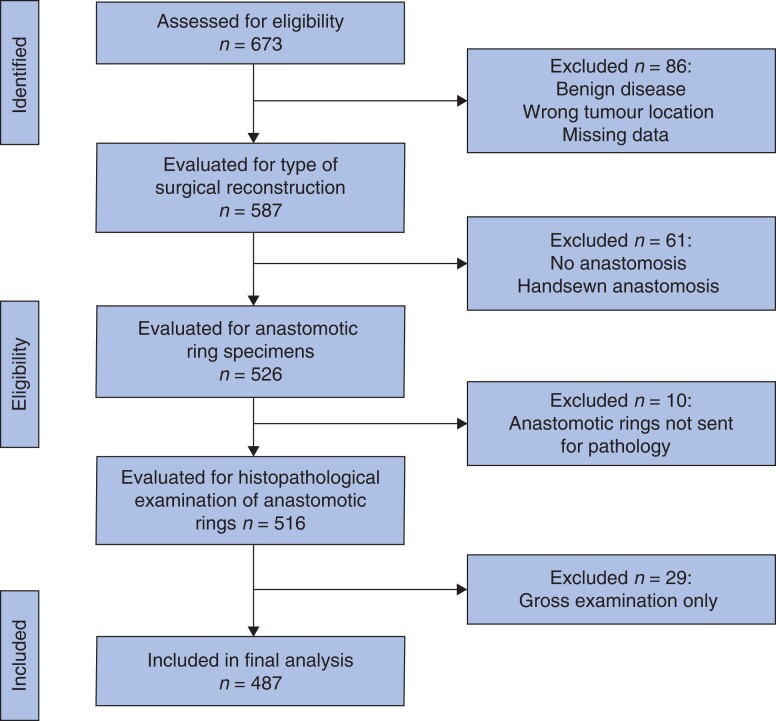
CONSORT flow diagram showing patient exclusions for retrospective study

A total of 673 patient records were evaluated for study inclusion, and 487 patients were identified as meeting the inclusion criteria (*Fig. 1*). Excluded patients were those who underwent permanent end colostomy, those without invasive malignancy, and those who did not have anastomotic rings sent for analysis based on factors such as palliative surgery or surgeon decision. Patient characteristics are listed in *Table 1*. All patients examined had a negative distal margin on the main surgical specimen. The completeness of TME was not reported in patients who had sigmoid colon cancers, or who had pathology reports from before routine description of this finding.

**Table 1:** Retrospective study patient characteristics

	*n* = 487
**Patient demographics**	
Age (years)	62.5 ± 12.2
Sex ratio (M:F)	314 (64.5%):173 (35.5%)
BMI (kg/m^2^)	28.0 ± 5.6
ASA grade	2.2 ± 0.6
Previous abdominal surgery	253 (51.9)
IBD/polyposis syndrome/Lynch syndrome	4 (0.8)
Family history of colorectal cancer	87 (17.9)
Neoadjuvant therapy	260 (53.3)
**Tumour characteristics**	
Pathological T category	
0	44 (9.0)
1	125 (25.6)
2	120 (24.6)
3	175 (35.9)
4	22 (4.5)
Tumour location	
Low rectum (≤6 cm)	73 (15.0)
Middle rectum (6–12 cm)	232 (47.6)
Upper rectum (12–15 cm)	65 (13.3)
Rectosigmoid (>15 cm)	65 (13.3)
Sigmoid	48 (10.0)
Repeat surgery, location unclear	3 (0.6)
Histology	
Adenocarcinoma	484 (99.4)
Neuroendocrine tumour	2 (0.4)
Gastrointestinal stromal tumour	1 (0.2)
**Surgery details**	
Surgical approach	
Open	313 (64.3)
Laparoscopic	148 (30.4)
Converted	26 (5.3)
Multi-visceral organ reconstruction	76 (15.6)
Distal margin status	
Negative	487 (100.0)
Positive	0 (0.0)
TME completeness	
Total recorded	198
Complete	154 (77.8)
Nearly complete	38 (19.2)
Incomplete	6 (3.0)

Values are *n* (%) unless otherwise indicated. IBD, inflammatory bowel disease; TME, total mesorectal excision.

*Table 2* shows the outcomes of interest for the microscopic pathological examination of anastomotic rings. A total of 14 proximal specimens and 21 distal specimens had a described pathological finding, across 25 total sets of anastomotic rings.

**Table 2:** Retrospective study anastomotic ring findings

Pathological exam		*n* = 487
	Microscopic for distal and proximal	479 (98.4)
	Gross for proximal, microscopic for distal	8 (1.6)
Proximal ring pathology findings		
	Absent	465 (95.5)
	Present	14 (2.9)
	Microscopic examination absent	8 (1.6)
Distal ring pathology findings		
	Absent	466 (95.7)
	Present	21 (4.3)
Pathological findings		
	Total	25
	Cancer	0
	Radiation changes	11
	Diverticula	8
	Tubular adenoma without HGD	2
	Ulceration	1
	Ischaemia	1
	HGD	1
	Hyperplastic polyp	1

Values are *n* (%) unless otherwise indicated. HGD, high-grade dysplasia.

None of the patients examined had findings of cancer within the anastomotic ring specimens. Twenty-five (5.1 per cent) patients had benign pathology findings in the anastomotic rings, most commonly inflammatory changes, hyperplastic polyps, and adenomatous polyps without high-grade dysplasia. Included in this number were 0.8 per cent of patients who had a benign neoplastic process. None of the patients had changes in clinical management related to the findings of their anastomotic ring specimens.

### Systematic review and meta-analysis

The electronic database search identified 5840 citations and manual searching identified eight citations. After duplicates were removed, 3730 records remained. Title and abstract screening determined that 246 studies were eligible for full-text review. Full-text review yielded a total of 27 articles meeting the specified inclusion criteria (*Fig. S1*)^2–13,22–36^. A total of 6861 patients were contained within these studies and 4368 of these patients had anastomotic rings sent for histopathological analysis. The effect of anastomotic ring findings on patient management was reported for 3054 patients across 19 studies. Distal margin status was reported in 13 studies. The operation performed was described in 24 publications. Out of 3902 listed operations, there were 3860 anterior resections/LARs (with inconsistent or absent distinctions between these procedures among various studies), 14 subtotal colectomies, 12 left hemicolectomies, and 16 unspecified procedures.

Cancer was reported in anastomotic ring specimens for 11 patients, always in the distal ring. Two studies changed management according to negative donut histopathology when main specimen distal margins were positive^27,30^. This management was contrary to other included studies and local practice. Post hoc sensitivity analysis was performed with and without these studies’ data censored to determine their effect on overall change in management rates. One author reported that patient management was affected in two of these patients after final pathology was reviewed, with both undergoing subsequent abdominoperineal resection^30^. Both patients had no residual cancer in the final specimen, though one suffered a locoregional recurrence 23 months later. Furthermore, in that same publication, 14 patients with a distal margin that was either positive or 1 mm or less had a negative anastomotic ring specimen used to confirm the decision against further surgery. Among other studies, none of the patients with cancer in the anastomotic ring specimens had treatment altered based on these findings. Six patients had positive margins on their main surgical specimen that determined their treatment course. Two had extensive metastatic disease either before surgery or soon after surgery, and one was felt to have a separate synchronous adenocarcinoma in the distal anastomotic ring that was resected by the EEA stapler firing. Another study did not report any cancer within anastomotic ring specimens but did describe an algorithm using anastomotic ring status for patients with a positive distal resection margin of the main colorectal cancer specimen^27^. Patients underwent either re-resection for a positive anastomotic ring (0 cases) or adjuvant chemoradiation for a negative anastomotic ring (69 cases). Based on the significant departure from standard management of colorectal cancer treatment described in these two publications, they were excluded from meta-analysis. Sensitivity analysis was performed, and calculations that include these two publications are displayed in *Fig. S2*.

**Fig. 2:**
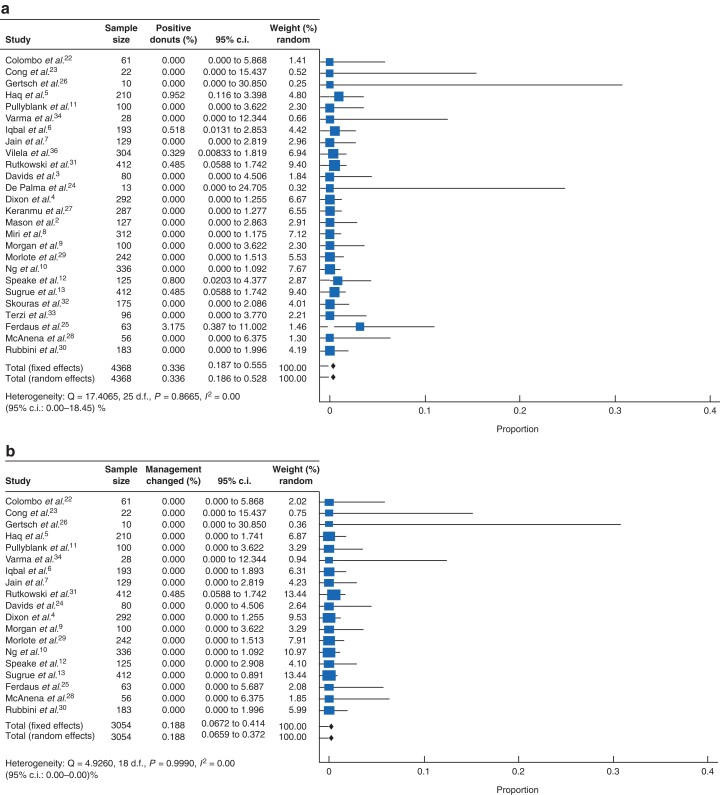
Forest plots of anastomotic ring specimens a) positive for cancer and b) altering patient management

Meta-analysis demonstrated an overall pooled frequency of cancer within anastomotic ring specimens of 0.34 per cent (95 per cent c.i. 0.19 to 0.53, 27 studies, 4368 participants, *I*^2^ = 0 per cent; *Fig. 2*). Anastomotic rings resulted in changed patient management in 0.19 per cent (95 per cent c.i. 0.066 to 0.37, 19 studies, 3054 participants, *I*^2^ 0 per cent). In sensitivity analysis taking a more liberal definition of altered patient management by including patients from publications with non-standard treatment regimens (negative donut with positive distal resection margin led to no surgery), there was a 0.72 per cent (95 per cent c.i. 0.039 to 2.25, 20 studies, 3341 patients, *I*^2^ = 92.62 per cent) frequency of altered patient management.

Finally, pooled analysis identified non-malignant anastomotic ring findings in a total of 74 patients (1.7 per cent). The most common pathologies included non-specific inflammatory change, hyperplastic polyps, and adenomatous polyps without high-grade dysplasia. These findings did not alter patient management in any instance.

#### Risk of bias

**Fig. 3:**
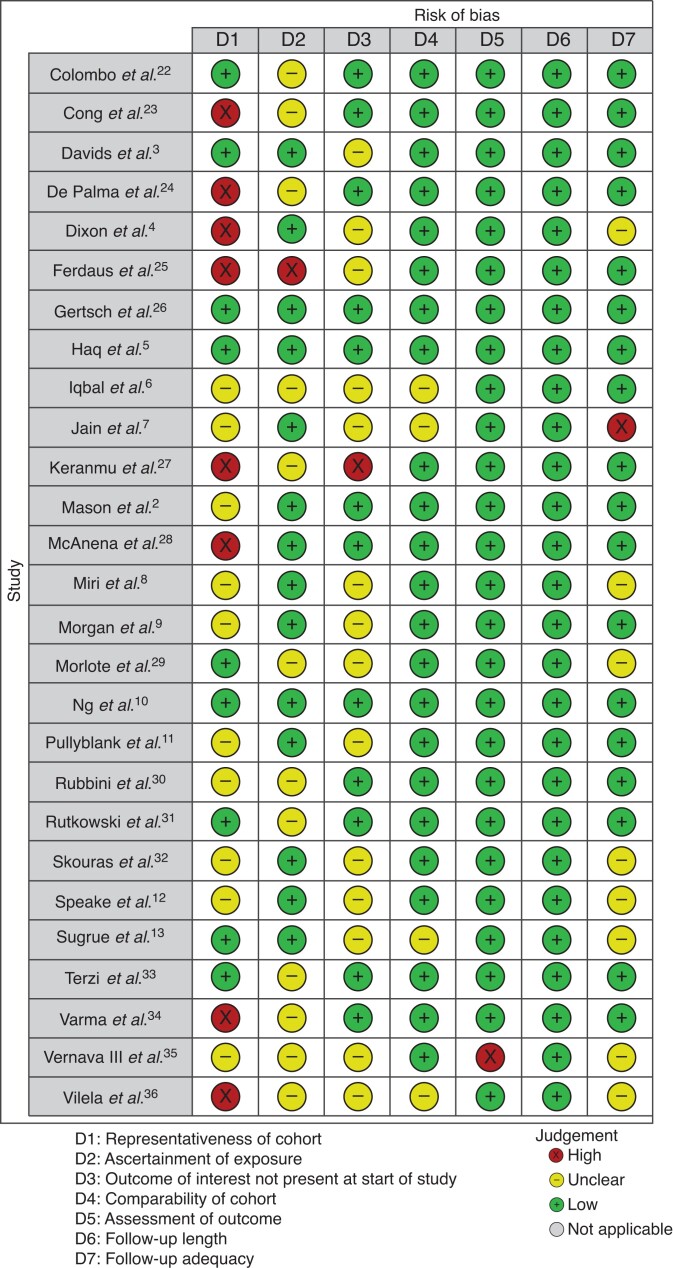
Risk-of-bias summary using modified Newcastle–Ottawa scale for systematic review

Risk-of-bias findings with respect to the primary outcome are summarized in *Fig. 3* and presented in detail in *Table S1*. All included publications were observational in nature. Only three studies had low risk of bias across all domains^5,10,26^. The remainder had unclear or high risk of bias, most commonly related to representativeness of the cohort to the general population.

## Discussion

In this retrospective study involving 487 patients undergoing circular stapler reconstruction after radical resection for colorectal cancer, there were no instances of cancer in the anastomotic ring specimens. Benign findings were present in 5.1 per cent of anastomotic rings (including benign neoplastic findings in 0.8 per cent of specimens) and did not change clinical management for any patients. This is the largest set of patient data on this topic to date, further supporting the notion that routine anastomotic ring histopathological analysis is an unnecessary practice. Additionally, it provides a uniquely Canadian patient cohort, which has not previously been specifically studied for this outcome. The proportion of patients who underwent laparoscopic surgery (30 per cent) is greater than in many other studies of this topic. The systematic literature review included 27 studies reporting anastomotic ring histopathology data on 6861 patients and found a very low frequency of cancer occurrences in the specimens, with most cases accompanied by positive main specimen margins. On pooled analysis, findings of cancer within the anastomotic ring specimens occurred in only 0.34 per cent of patients. The frequency with which these results affected patient management were even lower at 0.19 per cent.

Previously, another study also conducted a systematic review of anastomotic ring pathology, which included eight studies, 1754 patients, and concluded that the practice of routine pathological analysis of anastomotic rings should be re-evaluated^37^. In comparison, the systematic review presented here used a more comprehensive search strategy and retrieved a larger number of studies. Studies reporting anastomotic ring histopathological findings that were not specifically designed to address the question of their usefulness were identified, thus increasing the pool of patients available for meta-analysis and increasing the statistical power of the data.

Interestingly, the systematic review identified two studies that described practices inconsistent with conventional rectal cancer practice^22,23^. In both, the authors interpreted negative anastomotic rings as reassuring in the context of a positive main specimen distal margin, a practice that many rectal cancer surgeons would avoid. These two studies were excluded from the main analysis for determination of patient management change, as this practice was incongruent with standard of care and the remainder of the included studies. For practitioners who operate in settings where negative anastomotic rings are used as a negative distal margin, routine histopathological analysis of anastomotic rings might be marginally more useful; however, this practice is not commonplace. Anastomotic rings do not necessarily include a complete circumferential specimen of the bowel and therefore cannot be taken to reliably represent an additional resection margin^38^. In particular, if a circular stapler’s ‘spike’ is not brought out directly through the proximal end of the colorectal stump, little or none of the distal ring will represent an additional distal margin.

While the present study does not support the use of routine histopathological analysis of circular stapler anastomotic rings after resection of colorectal malignancy, there may be exceptions where directed analysis is prudent. In cases where the surgeon has a compelling clinical reason to believe that the anastomotic ring might have some meaningful finding, histopathological analysis could be considered selectively. Otherwise, the cost of pathological analysis has been reported in several studies, including figures up to $643 USD (€631)^9–11,13^. Alleviating the burden of the pathologist’s time and effort as well as expenses for healthcare systems would be immensely beneficial.

This study has several limitations. One drawback of both the local study and systematic review is the retrospective nature of the data and absence of a comparator patient group. Without randomization, patients who had anastomotic rings submitted for pathology may have been subject to selection bias. Additionally, the local study involved high-volume colorectal surgeons, whose patient population and outcomes may not be reflective of all surgeons who use EEA staplers; however, a prospective randomized clinical trial addressing this topic is unlikely to occur and is also unlikely to provide new information due to the low frequency of events. Another limitation, unique to the systematic review, is the overall quality of the included studies. Most had at least an unclear risk of bias due to poor methods reporting. While meta-analyses can increase power and precision, they cannot eliminate any biases that exist in pooled data. Another limitation is that several studies did not report whether anastomotic ring findings changed patient management; however, of those six studies that did not comment on changes to management, none reported anastomotic rings that were positive for cancer on histopathology. Last, the rate of patient management change may have been overestimated in the meta-analysis due to reporting bias where studies with positive findings might be more likely to describe this outcome.

## Supplementary Material

zrac122_Supplementary_Data

## Data Availability

The data that support the finding of this study are available from the corresponding author, EH, upon reasonable request.
