# Supplementary material for: Routine pathologic evaluation of circular stapler anastomotic rings is not useful after resection for colorectal cancer: retrospective study and systematic review with meta-analysis
Source: BJS Open. 2022 Oct 12;6(5):zrac122. doi: 10.1093/bjsopen/zrac122 (PMC9553864; doi:10.1093/bjsopen/zrac122)
Supplement: zrac122_Supplementary_Data [file zrac122_supplementary_data.docx]

**Routine Pathologic Evaluation of Circular Stapler Anastomotic Rings is Not Useful after Resection for Colorectal Cancer: Retrospective Study and Systematic Review with Meta-Analysis**

**Authors:**

James R. Holden, M.D.^1^

Pam McIntosh, M.D.^1^

Garrett G. R. J. Johnson, M.D.^1,2^

Jason Park, M.D. M.Ed.^3^

David J. Hochman, M.D.^1^

Ashley Vergis, M.D.^1^

Benson Yip, M.D.^1^

Ramzi M. Helewa, M.D. M.Sc.^1^

Eric Hyun, M.D., Ph.D.^1^

1 - Department of Surgery, University of Manitoba and St. Boniface Hospital, Winnipeg, Manitoba, Canada

2 – Clinician investigator program, University of Manitoba

3 – Department of Surgery, University of British Columbia, Vancouver, British Columbia, Canada

**Correspondence:**

Eric Hyun, M.D., Ph.D.

St. Boniface Hospital, Z3037 – 409 Taché Avenue, Winnipeg, Manitoba, Canada, R2H 2A6

Tel: 1-204-235-3900, Fax: 1-204-237-8141, Email: [ehyun@sbgh.mb.ca](mailto:ehyun@sbgh.mb.ca)

**Index:**

| Table S1: Risk of bias assessments for individual studies in systematic review | pag 2 |
| --- | --- |
| Figure S1: PRISMA Diagram for systematic review | pag 15 |
| Figure S2: Meta-analysis of cases where anastomotic rings changed management, including where management was inconsistent with recommended practice in systematic review | pag 16 |
| References | pag 17 |

**Table S1:** Risk of bias assessments for individual studies in systematic review

*Colombo, 1987*

| **Bias Type** | **Judgment** | **Commentary** |
| --- | --- | --- |
| Representativeness of Cohort (Selection) | Low risk | Single institution retrospective cohort with patient and tumor characteristics clearly outlined |
| Ascertainment of Exposure (Selection) | Unclear risk | How patients were identified is not clearly outlined |
| Outcome Absent at Start of Study (Selection) | Low risk | All anastomotic rings sent for histopathologic analysis |
| Comparability of Cohort (Comparability) | Low risk | No stratification but no events in this patient group |
| Assessment of Outcome (Outcome) | Low risk | Pathology records |
| Follow-up Length (Outcome) | Low risk | Anastomotic ring pathology available with no events, so no further follow-up required |
| Follow-up Adequacy (Outcome) | Low risk | All anastomotic rings accounted for |

*Cong, 2014*

| **Bias Type** | **Judgment** | **Commentary** |
| --- | --- | --- |
| Representativeness of Cohort (Selection) | High risk | Prospective cohort of highly selected patients with colorectal cancer for laparoscopic ISR |
| Ascertainment of Exposure (Selection) | Unclear risk | How patients were identified is not clearly outlined |
| Outcome Absent at Start of Study (Selection) | Low risk | All anastomotic rings sent for frozen section and pathologic analysis |
| Comparability of Cohort (Comparability) | Low risk | No stratification but no events in this patient group |
| Assessment of Outcome (Outcome) | Low risk | Frozen section and formal histopathology records |
| Follow-up Length (Outcome) | Low risk | Anastomotic ring pathology available with no events, so no further follow-up required |
| Follow-up Adequacy (Outcome) | Low risk | All anastomotic rings accounted for |

*Davids, 2014*

| **Bias Type** | **Judgment** | **Commentary** |
| --- | --- | --- |
| Representativeness of Cohort (Selection) | Low risk | Single institution retrospective cohort with patient and tumor characteristics clearly outlined |
| Ascertainment of Exposure (Selection) | Low risk | Institution’s tumor registry used |
| Outcome Absent at Start of Study (Selection) | Unclear | Two anastomotic ring sets not sent for pathology, unclear why (ex. Macroscopically apparent positive distal margin) |
| Comparability of Cohort (Comparability) | Low risk | No stratification but no events in this patient group |
| Assessment of Outcome (Outcome) | Low risk | Pathology records from electronic medical records |
| Follow-up Length (Outcome) | Low risk | Anastomotic ring pathology available with no events, 30 months of follow-up recorded nonetheless |
| Follow-up Adequacy (Outcome) | Low risk | <3% of anastomotic rings unaccounted for |

*De Palma, 2014*

| **Bias Type** | **Judgment** | **Commentary** |
| --- | --- | --- |
| Representativeness of Cohort (Selection) | High risk | Prospective cohort, study included only patients with no neoadjuvant therapy or IBD |
| Ascertainment of Exposure (Selection) | Unclear risk | How patients were identified is not clearly outlined |
| Outcome Absent at Start of Study (Selection) | Low risk | All anastomotic rings sent for histopathologic analysis |
| Comparability of Cohort (Comparability) | Low risk | No stratification but no events in this patient group |
| Assessment of Outcome (Outcome) | Low risk | Pathologic analysis of anastomotic rings |
| Follow-up Length (Outcome) | Low risk | Anastomotic ring pathology available with no events, so no further follow-up required |
| Follow-up Adequacy (Outcome) | Low risk | All anastomotic rings accounted for |

*Dixon, 2020*

| **Bias Type** | **Judgment** | **Commentary** |
| --- | --- | --- |
| Representativeness of Cohort (Selection) | High risk | Retrospective cohort data missing some demographic information, also included many non-cancer cases |
| Ascertainment of Exposure (Selection) | Low risk | Patient identification through coding department using case notes and pathology reports |
| Outcome Absent at Start of Study (Selection) | Unclear risk | Only 292 patients had anastomotic rings sent for analysis, unclear why (ex. Macroscopically apparent positive distal margin, benign disease, etc.) |
| Comparability of Cohort (Comparability) | Low risk | No stratification but no events in this patient group |
| Assessment of Outcome (Outcome) | Low risk | Pathology reports of anastomotic rings |
| Follow-up Length (Outcome) | Low risk | Anastomotic ring pathology available with no events, so no further follow-up required |
| Follow-up Adequacy (Outcome) | Unclear risk | 17% of anastomotic rings not sent to pathology department, without clear explanation why |

*Ferdaus, 2020*

| **Bias Type** | **Judgment** | **Commentary** |
| --- | --- | --- |
| Representativeness of Cohort (Selection) | High risk | Purposive sampling method of patients undergoing stapled reconstruction for low rectal cancer |
| Ascertainment of Exposure (Selection) | High risk | Details of purposive sampling and how cases selected not clearly outlined |
| Outcome Absent at Start of Study (Selection) | Unclear risk | Not clear whether sampling method preferentially chose patients with outcome of interest |
| Comparability of Cohort (Comparability) | Low risk | Patients stratified by distal anastomotic ring positivity during analysis |
| Assessment of Outcome (Outcome) | Low risk | Pathology reports and medical records |
| Follow-up Length (Outcome) | Low risk | Anastomotic ring pathology available with no events, so no further follow-up required |
| Follow-up Adequacy (Outcome) | Low risk | All anastomotic rings accounted for |

*Gertsch, 1992*

| **Bias Type** | **Judgment** | **Commentary** |
| --- | --- | --- |
| Representativeness of Cohort (Selection) | Low risk | Consecutive prospective sample of patients undergoing surgery with EEA stapling for rectal or rectosigmoid cancer |
| Ascertainment of Exposure (Selection) | Low risk | Patients prospectively enrolled and all accounted for via medical record data |
| Outcome Absent at Start of Study (Selection) | Low risk | All patients had anastomotic rings sent for pathology |
| Comparability of Cohort (Comparability) | Low risk | No stratification but no events in this patient group |
| Assessment of Outcome (Outcome) | Low risk | Pathologic report data for anastomotic rings |
| Follow-up Length (Outcome) | Low risk | Anastomotic ring pathology available with no events, so no further follow-up required |
| Follow-up Adequacy (Outcome) | Low risk | All anastomotic rings accounted for |

*Haq, 2020*

| **Bias Type** | **Judgment** | **Commentary** |
| --- | --- | --- |
| Representativeness of Cohort (Selection) | Low risk | All patients undergoing colorectal cancer surgery with EEA stapling |
| Ascertainment of Exposure (Selection) | Low risk | Identification from prospectively maintained local database via medical records |
| Outcome Absent at Start of Study (Selection) | Low risk | All patients had anastomotic rings sent for pathology |
| Comparability of Cohort (Comparability) | Low risk | Patients stratified by rectal specimen distal margin positivity during analysis |
| Assessment of Outcome (Outcome) | Low risk | Pathology report and medical record data of anastomotic rings |
| Follow-up Length (Outcome) | Low risk | Anastomotic ring pathology available with no events, so no further follow-up required |
| Follow-up Adequacy (Outcome) | Low risk | All anastomotic rings accounted for |

*Iqbal, 2017*

| **Bias Type** | **Judgment** | **Commentary** |
| --- | --- | --- |
| Representativeness of Cohort (Selection) | Unclear risk | Not explicitly stated whether all patients had colorectal cancer, minimal patient demographic information available, no inclusion/exclusion criteria available on patient sample |
| Ascertainment of Exposure (Selection) | Unclear risk | Retrospective analysis of histopathology results with no explanation of how patients were identified |
| Outcome Absent at Start of Study (Selection) | Unclear risk | Some anastomotic ring data unavailable for analysis, no clear explanation why |
| Comparability of Cohort (Comparability) | Unclear risk | No stratification of patients performed |
| Assessment of Outcome (Outcome) | Low risk | Hospital electronic database pathology data |
| Follow-up Length (Outcome) | Low risk | Anastomotic ring data available for adequate length to assess primary outcome |
| Follow-up Adequacy (Outcome) | Low risk | <10% of anastomotic rings unaccounted for, though secondary outcomes for patient with cancer in distal anastomotic ring not reported |

*Jain, 2012*

| **Bias Type** | **Judgment** | **Commentary** |
| --- | --- | --- |
| Representativeness of Cohort (Selection) | Unclear risk | Cohort of patients with rectal cancers below peritoneal reflection, minimal demographic data available |
| Ascertainment of Exposure (Selection) | Low risk | Patients collected from prospective clinicopathologic database |
| Outcome Absent at Start of Study (Selection) | Unclear risk | 42% of anastomotic rings sent for pathology without explanation of how these were selected |
| Comparability of Cohort (Comparability) | Unclear risk | Some stratification done based on distal specimen margin length but details lacking |
| Assessment of Outcome (Outcome) | Low risk | Pathologic analysis records from database |
| Follow-up Length (Outcome) | Low risk | Anastomotic ring pathology available with no events, so no further follow-up required |
| Follow-up Adequacy (Outcome) | High risk | >50% of anastomotic rings not analyzed, with varied indications for not being sent |

*Keranmu, 2018*

| **Bias Type** | **Judgment** | **Commentary** |
| --- | --- | --- |
| Representativeness of Cohort (Selection) | High risk | Single institution data on patients demonstrating positive distal resection margin of specimen and adjuvant treatment algorithm that are both incompatible with usual rectal cancer care in most parts of the world |
| Ascertainment of Exposure (Selection) | Unclear risk | Patient identification for study inclusion not explained |
| Outcome Absent at Start of Study (Selection) | High risk | Patient selection not explained and, in particular because of high distal margin positivity, unclear whether patients specially selected for potential to have positive anastomotic ring pathology |
| Comparability of Cohort (Comparability) | Low risk | Stratification by multiple variables (including distal margin status) performed during data analysis |
| Assessment of Outcome (Outcome) | Low risk | Pathologic analysis of anastomotic rings |
| Follow-up Length (Outcome) | Low risk | Anastomotic ring pathology available with no events, 66 months of regular follow-up recorded nonetheless |
| Follow-up Adequacy (Outcome) | Low risk | All anastomotic rings accounted for |

*Mason, 2014*

| **Bias Type** | **Judgment** | **Commentary** |
| --- | --- | --- |
| Representativeness of Cohort (Selection) | Unclear risk | Abstract only, unclear patient demographics |
| Ascertainment of Exposure (Selection) | Low risk | Patients selected based on chart audit using electronic medical records and histopathology reports |
| Outcome Absent at Start of Study (Selection) | Low risk | All patients had anastomotic rings sent for pathology |
| Comparability of Cohort (Comparability) | Low risk | No stratification but no events in this patient group |
| Assessment of Outcome (Outcome) | Low risk | Histopathology report results for anastomotic rings |
| Follow-up Length (Outcome) | Low risk | Anastomotic ring pathology available with no events, so no further follow-up required |
| Follow-up Adequacy (Outcome) | Low risk | All anastomotic rings accounted for |

*McAnena, 1990*

| **Bias Type** | **Judgment** | **Commentary** |
| --- | --- | --- |
| Representativeness of Cohort (Selection) | High risk | Single surgeon data, no neoadjuvant therapy included, only rectal cancers <7cm measured, other demographic details absent |
| Ascertainment of Exposure (Selection) | Low risk | Audit of patient medical records |
| Outcome Absent at Start of Study (Selection) | Low risk | All patients had anastomotic rings sent for pathology |
| Comparability of Cohort (Comparability) | Low risk | No stratification but no events in this patient group |
| Assessment of Outcome (Outcome) | Low risk | Independent assessment of pathology results by two authors |
| Follow-up Length (Outcome) | Low risk | Anastomotic ring pathology available with no events, so no further follow-up required |
| Follow-up Adequacy (Outcome) | Low risk | All anastomotic rings accounted for |

*Miri, 2004*

| **Bias Type** | **Judgment** | **Commentary** |
| --- | --- | --- |
| Representativeness of Cohort (Selection) | Unclear risk | Abstract only, patients undergoing anterior resection for colorectal cancer but other details of inclusion criteria missing |
| Ascertainment of Exposure (Selection) | Low risk | Patients identified from institutional colorectal cancer database |
| Outcome Absent at Start of Study (Selection) | Unclear risk | Some anastomotic rings not sent for pathology, unclear why |
| Comparability of Cohort (Comparability) | Low risk | Results stratified by distance to anal margin, and no events reported |
| Assessment of Outcome (Outcome) | Low risk | Histopathology report results for anastomotic rings |
| Follow-up Length (Outcome) | Low risk | Anastomotic ring pathology available with no events (save 4 metaplastic polyps), so no further follow-up required |
| Follow-up Adequacy (Outcome) | Unclear risk | >30% of cases did not have anastomotic rings sent for pathology, with no explanation of indications for this |

*Morgan, 2006*

| **Bias Type** | **Judgment** | **Commentary** |
| --- | --- | --- |
| Representativeness of Cohort (Selection) | Unclear risk | All patients undergoing surgery for colorectal cancer with EEA stapling where anastomotic rings sent for pathology, lacking demographic information |
| Ascertainment of Exposure (Selection) | Low risk | Patients identified from prospectively collected departmental database |
| Outcome Absent at Start of Study (Selection) | Unclear risk | Unclear if anastomotic rings were being selectively sent or not based on anticipated result per surgeon judgment |
| Comparability of Cohort (Comparability) | Low risk | Results stratified by distal resection margin length, and no events reported |
| Assessment of Outcome (Outcome) | Low risk | Histopathology reports and histology records both used |
| Follow-up Length (Outcome) | Low risk | Anastomotic ring pathology available with no events, so no further follow-up required |
| Follow-up Adequacy (Outcome) | Low risk | All anastomotic rings accounted for |

*Morlote, 2015*

| **Bias Type** | **Judgment** | **Commentary** |
| --- | --- | --- |
| Representativeness of Cohort (Selection) | Low risk | All patients undergoing surgery for a primary colorectal cancer |
| Ascertainment of Exposure (Selection) | Unclear risk | Method of identifying patient population not elaborated |
| Outcome Absent at Start of Study (Selection) | Unclear risk | Many patients in cohort did not have anastomotic rings sent, reasons why unclear |
| Comparability of Cohort (Comparability) | Low risk | No stratification but no events in this patient group |
| Assessment of Outcome (Outcome) | Low risk | Review of pathology reports |
| Follow-up Length (Outcome) | Low risk | Anastomotic ring pathology available with no events, so no further follow-up required |
| Follow-up Adequacy (Outcome) | Unclear risk | >50% of cases did not have anastomotic rings sent for pathology, with no explanation of indications for this |

*Ng, 2014*

| **Bias Type** | **Judgment** | **Commentary** |
| --- | --- | --- |
| Representativeness of Cohort (Selection) | Low risk | All cases undergoing colorectal cancer surgery |
| Ascertainment of Exposure (Selection) | Low risk | Patients selected from prospective colorectal cancer database |
| Outcome Absent at Start of Study (Selection) | Low risk | All patients had anastomotic rings sent for pathology with specific statement from authors that this is done routinely |
| Comparability of Cohort (Comparability) | Low risk | Results stratified by distal margin length, and no events reported |
| Assessment of Outcome (Outcome) | Low risk | Review of histology reports |
| Follow-up Length (Outcome) | Low risk | Anastomotic ring pathology available with no events, so no further follow-up required |
| Follow-up Adequacy (Outcome) | Low risk | All anastomotic rings accounted for |

*Pullyblank, 2001*

| **Bias Type** | **Judgment** | **Commentary** |
| --- | --- | --- |
| Representativeness of Cohort (Selection) | Unclear risk | Anastomotic rings from a single institution, patient population not specified |
| Ascertainment of Exposure (Selection) | Low risk | Identified via patient medical records through a computerized pathology system |
| Outcome Absent at Start of Study (Selection) | Unclear risk | Lacking description of how it is decided whether anastomotic rings are sent for pathology |
| Comparability of Cohort (Comparability) | Low risk | No stratification but no events in this patient group |
| Assessment of Outcome (Outcome) | Low risk | Review of histology reports and patient medical records |
| Follow-up Length (Outcome) | Low risk | Anastomotic ring pathology available with no events, so no further follow-up required |
| Follow-up Adequacy (Outcome) | Low risk | All anastomotic rings accounted for |

*Rubbini, 1990*

| **Bias Type** | **Judgment** | **Commentary** |
| --- | --- | --- |
| Representativeness of Cohort (Selection) | Unclear risk | Included all patients undergoing LAR at one institution, but excluded any patients without long-term follow-up data |
| Ascertainment of Exposure (Selection) | Unclear risk | Identified via patient medical records and histopathology reports |
| Outcome Absent at Start of Study (Selection) | Low risk | All patients had anastomotic rings sent for pathology with specific statement from authors that this is done routinely |
| Comparability of Cohort (Comparability) | Low risk | No stratification but no events in this patient group |
| Assessment of Outcome (Outcome) | Low risk | Review of histopathology reports |
| Follow-up Length (Outcome) | Low risk | Anastomotic ring pathology available with no events, so no further follow-up required |
| Follow-up Adequacy (Outcome) | Low risk | All anastomotic rings accounted for |

*Rutkowski, 2011*

| **Bias Type** | **Judgment** | **Commentary** |
| --- | --- | --- |
| Representativeness of Cohort (Selection) | Low risk | All patients with rectal cancer and negative CRM during study period |
| Ascertainment of Exposure (Selection) | Unclear risk | Use of patient medical records specified, but no more specific indication of how patients identified |
| Outcome Absent at Start of Study (Selection) | Low risk | Clear description of inclusion criteria, all anastomotic rings sent for pathology |
| Comparability of Cohort (Comparability) | Low risk | Results stratified by distal margin status |
| Assessment of Outcome (Outcome) | Low risk | Histopathology reports and patient medical records |
| Follow-up Length (Outcome) | Low risk | Adequate length of patient follow-up, median 75 months, including description of both patients with positive anastomotic rings |
| Follow-up Adequacy (Outcome) | Low risk | All anastomotic rings accounted for |

*Skouras, 2014*

| **Bias Type** | **Judgment** | **Commentary** |
| --- | --- | --- |
| Representativeness of Cohort (Selection) | Unclear risk | Includes all patients having surgery for colorectal cancer but specific demographic details lacking |
| Ascertainment of Exposure (Selection) | Low risk | Identified via prospective colorectal cancer database |
| Outcome Absent at Start of Study (Selection) | Unclear risk | Criteria for sending anastomotic rings for pathology not outlined, unclear whether rings analyzed routinely or only on basis of anticipated outcome |
| Comparability of Cohort (Comparability) | Low risk | No events in this patient group, distal margin positivity reported |
| Assessment of Outcome (Outcome) | Low risk | Data from histopathology reports |
| Follow-up Length (Outcome) | Low risk | Anastomotic ring pathology available with no events, so no further follow-up required |
| Follow-up Adequacy (Outcome) | Unclear risk | >50% of cases did not have anastomotic rings sent for pathology, with no explanation of indications for this |

*Speake, 2003*

| **Bias Type** | **Judgment** | **Commentary** |
| --- | --- | --- |
| Representativeness of Cohort (Selection) | Unclear risk | Includes patients undergoing surgery for rectal adenocarcinoma but specific demographic details lacking |
| Ascertainment of Exposure (Selection) | Low risk | Identified using a gastrointestinal cancer database |
| Outcome Absent at Start of Study (Selection) | Unclear risk | Criteria for sending anastomotic rings for pathology not outlined, unclear whether rings analyzed routinely or only on basis of anticipated outcome |
| Comparability of Cohort (Comparability) | Low risk | No stratification but no events in this patient group |
| Assessment of Outcome (Outcome) | Low risk | Data from histopathology reports and patient medical records |
| Follow-up Length (Outcome) | Low risk | Anastomotic ring pathology available with no events, so no further follow-up required |
| Follow-up Adequacy (Outcome) | Unclear risk | >50% of cases did not have anastomotic rings sent for pathology, with no explanation of indications for this |

*Sugrue, 2017*

| **Bias Type** | **Judgment** | **Commentary** |
| --- | --- | --- |
| Representativeness of Cohort (Selection) | Low risk | Multi-center cohort of patients undergoing surgery for rectal and rectosigmoid cancers using EEA staple |
| Ascertainment of Exposure (Selection) | Low risk | Identified using prospective colorectal cancer database |
| Outcome Absent at Start of Study (Selection) | Unclear risk | Criteria for sending anastomotic rings for pathology not outlined, unclear whether rings analyzed routinely or only on basis of anticipated outcome |
| Comparability of Cohort (Comparability) | Unclear risk | Anastomotic ring pathology not stratified according to other variables |
| Assessment of Outcome (Outcome) | Low risk | Data from electronic medical record pathology reports |
| Follow-up Length (Outcome) | Low risk | Anastomotic ring pathology available with no events, so no further follow-up required |
| Follow-up Adequacy (Outcome) | Unclear risk | 15% of cases did not have anastomotic rings sent for pathology, with no explanation of indications for this |

*Terzi, 2006*

| **Bias Type** | **Judgment** | **Commentary** |
| --- | --- | --- |
| Representativeness of Cohort (Selection) | Low risk | Patients undergoing surgery with EEA stapling for rectal and distal sigmoid cancers |
| Ascertainment of Exposure (Selection) | Unclear risk | No clear description of how patients were identified |
| Outcome Absent at Start of Study (Selection) | Low risk | Anastomotic rings analyzed in all patients |
| Comparability of Cohort (Comparability) | Low risk | No stratification but no events in this patient group |
| Assessment of Outcome (Outcome) | Low risk | Data from histopathology reports |
| Follow-up Length (Outcome) | Low risk | Anastomotic ring pathology available with no events, so no further follow-up required |
| Follow-up Adequacy (Outcome) | Low risk | All anastomotic rings accounted for |

*Varma, 1990*

| **Bias Type** | **Judgment** | **Commentary** |
| --- | --- | --- |
| Representativeness of Cohort (Selection) | High risk | Patients undergoing surgery below peritoneal reflection, not all with cancer, lacking demographic details |
| Ascertainment of Exposure (Selection) | Unclear risk | No clear description of how patients were identified |
| Outcome Absent at Start of Study (Selection) | Low risk | Anastomotic rings analyzed in all patients |
| Comparability of Cohort (Comparability) | Low risk | No stratification but no events in this patient group |
| Assessment of Outcome (Outcome) | Low risk | Data from histopathology reports |
| Follow-up Length (Outcome) | Low risk | Anastomotic ring pathology available with no events, so no further follow-up required |
| Follow-up Adequacy (Outcome) | Low risk | All anastomotic rings accounted for |

*Vernava III, 1992*

| **Bias Type** | **Judgment** | **Commentary** |
| --- | --- | --- |
| Representativeness of Cohort (Selection) | Unclear risk | Patients undergoing curative colorectal cancer resection, but excluded any patients with follow-up less than 1 year |
| Ascertainment of Exposure (Selection) | Unclear risk | No clear description of how patients were identified |
| Outcome Absent at Start of Study (Selection) | Unclear risk | Criteria for sending anastomotic rings for pathology not outlined, unclear whether rings analyzed routinely or only on basis of anticipated outcome |
| Comparability of Cohort (Comparability) | Low risk | No stratification but no events in this patient group |
| Assessment of Outcome (Outcome) | High risk | Unclear reporting of how many anastomotic rings were examined |
| Follow-up Length (Outcome) | Low risk | Anastomotic ring pathology available with no events, so no further follow-up required |
| Follow-up Adequacy (Outcome) | Unclear risk | Unclear reporting of how many anastomotic rings were examined |

*Vilela, 2018*

| **Bias Type** | **Judgment** | **Commentary** |
| --- | --- | --- |
| Representativeness of Cohort (Selection) | High risk | Patients undergoing LAR for any neoplastic process with the use of an anastomotic stapler, lacking description of inclusion and exclusion criteria |
| Ascertainment of Exposure (Selection) | Unclear risk | No clear description of how patients were identified |
| Outcome Absent at Start of Study (Selection) | Unclear risk | Criteria for sending anastomotic rings for pathology not outlined, unclear whether rings analyzed routinely or only on basis of anticipated outcome |
| Comparability of Cohort (Comparability) | Unclear risk | Anastomotic ring pathology not stratified according to other variables |
| Assessment of Outcome (Outcome) | Low risk | Data from histopathology reports |
| Follow-up Length (Outcome) | Low risk | Anastomotic ring pathology available with no events, median 22 months of regular follow-up recorded nonetheless |
| Follow-up Adequacy (Outcome) | Unclear risk | Appears that all anastomotic rings accounted for but reported as percentages only rather than patient numbers |

**Figure S1:** PRISMA Diagram for systematic review

**
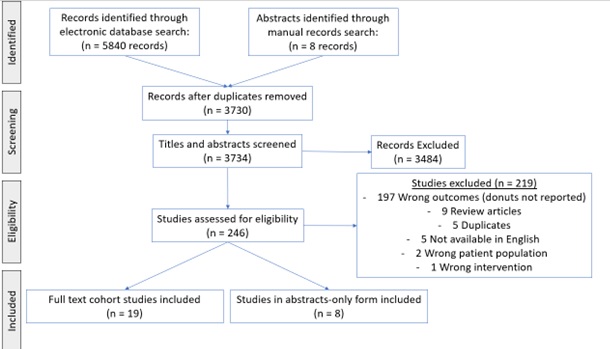
**

**Figure S2:** Meta-analysis of cases where anastomotic rings changed management, including where management was inconsistent with recommended practice in systematic review

**
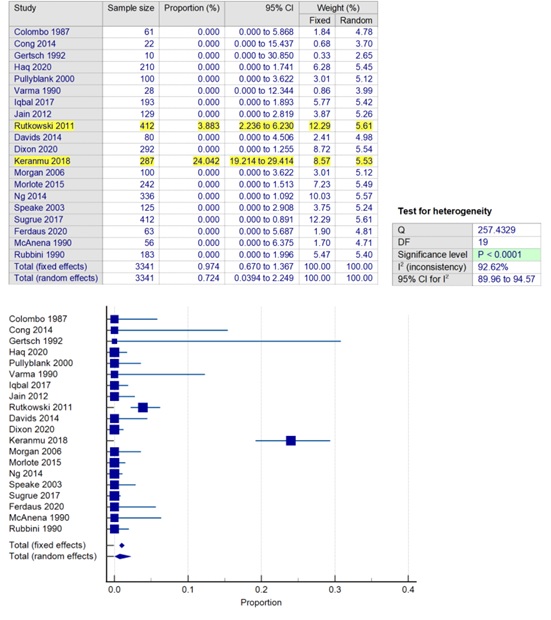
**

**References**

1. Kyzer S, Gordon PH. Experience with the use of the circular stapler in rectal surgery. Dis Colon Rectum. 1992;35(7):696-706.
2. Mason C, Przybyl K, Brelsford K. Histopathological assessment of ‘doughnuts’ after anterior resection. A regional survey and single institution audit. Colorectal Dis. 2014;16(Suppl 2):151-2.
3. Davids J, Cherng N, Sturrock P, et al. Time to toss the donuts? A pilot study of EEA stapler anastomotic ring pathology in rectal cancer cases. Dis Colon Rectum. 2014;57(5):e330.
4. Dixon S, Barrow H, Hughes J. Doughnut bother! Histopathological examination of anastomotic doughnuts following colorectal anastomosis does not change patient management. Surg Exp Pathol. 2020;3(1)1-4.
5. Haq I, Shakeel O, Amjad A, et al. Benefits of Outcomes of the Microscopic Examination of Anastomotic Donuts After Colorectal Resection for Oncological Purposes: A Medical Record-Based Study. Cureus. 2020;12(5):e7932.
6. Iqbal MR, Deputy M, Bailey S, Lawes D. Routine ‘doughnut’ histology after anterior resection: Does it add any clinical benefit?. Colorectal Disease. 2017;19(Suppl 2):97.
7. Jain P, Saad A, Bowley D, Karandikar S. Should we analyse the doughnuts after stapled restorative surgery for low rectal cancer?. Colorectal Dis. 2012;14(Suppl 2):9.
8. Miri B, Joondan Z, Kelly M. Routine histological examination of “doughnuts” following anterior resection for rectal cancers: is it a fruitless exercise?. Brit J Surg. 2004;91(Suppl):3.
9. Morgan A, Dawson PM, Smith JJ. Histological examination of circular stapled ‘doughnuts’: questionable routine practice?. Surgeon 2006;4(2):75-7.
10. Ng CW, Lieske B, Tan KK. Routine histological sampling of doughnuts post oncologic anterior resection is not necessary. Int J Colorectal Dis. 2014;29(7):843-5.
11. Pullyblank AM, Kirwan C, Rigby HS, Dixon AR. Is routine histological reporting of doughnuts justified after anterior resection for colorectal cancer?. Colorectal Dis. 2001;3(3):198-200.
12. Speake WJ, Abercrombie JF. Should 'doughnut' histology be routinely performed following anterior resection for rectal cancer?. Ann R Coll Surg Engl. 2003;85(1):26.
13. Sugrue J, Dagbert F, Park J, et al. No clinical benefit from routine histologic examination of stapler doughnuts at low anterior resection for rectal cancer. Surgery. 2017;162(1):147-51.
14. Loughrey MB, Quirke P, Shepherd NA. Standards and datasets for reporting cancers: Dataset for histopathological reporting of colorectal cancer, September 2018. Royal College of Pathologists. 2018.
15. Von Elm E, Altman DG, Egger M, et al. The Strengthening the Reporting of Observational Studies in Epidemiology (STROBE) statement: guidelines for reporting observational studies. Lancet. 2007; 370(9596):1453-7.
16. Chandler J, Churchill R, Higgins J, et al. Methodological standards for the conduct of new Cochrane Intervention Reviews (MECIR). Methodological Standard for the Conduct of New Cochrane Intervention Reviews. 2013.
17. Liberati A, Douglas G, Altman J, et al. The PRISMA statement for reporting systematic reviews and meta-analyses of studies that evaluate health care interventions: explanation and elaboration. J Clin Epidemiol. 2009;62(10):e1-34.
18. Fain SN, Patin CS, Morgenstern L. Use of a mechanical suturing apparatus in low colorectal anastomosis. Arch Surg. 1975;110(9):1079-82.
19. Lewis-Lloyd C, Adiamah A, Pettitt E, Crooks C, Humes D. Risk of post-operative venous thromboembolism after surgery for colorectal malignancy: A systematic review and meta-analysis. Dis Colon Rectum. 2021;64(4):484-96.
20. Freeman MF, Tukey JW. Transformations related to the angular and the square root. Ann Math Stat. 1950;21(4):607-11.
21. Higgins JP, Thompson SG. Quantifying heterogeneity in a meta-analysis. Stat Med. 2002;21(11):1539-58.
22. Colombo PL, Foglieni CL, Morone C. Analysis of recurrence following curative low anterior resection and stapled anastomoses for carcinoma of the middle third and lower rectum. Dis Colon Rectum. 1987;30(6):457-64.
23. Cong JC, Chen CS, Ma MX, Xia ZX, Liu DS, Zhang FY. Laparoscopic intersphincteric resection for low rectal cancer: comparison of stapled and manual coloanal anastomosis. Colorectal Dis. 2014;16(5):353-8.
24. De Palma GD, Luglio G, Staibano S, et al. Perioperative characterization of anastomotic doughnuts with high-resolution probe-based confocal laser endomicroscopy in colorectal cancer surgery: a feasibility study. Surg Endosc. 2014;28(7):2072-7.
25. Ferdaus AM, Hossain MS, Islam SH, et al. The role of histological assessment of distal doughnut in low anterior resection for low rectal cancer. Mymensingh Med J. 2020;29(1):73-7.
26. Gertsch P, Baer HU, Kraft R, Maddern GJ, Altermatt HJ. Malignant cells are collected on circular staplers. Dis Colon Rectum. 1992;35(3):238-41.
27. Keranmu A, Liu HN, Wu YC, et al. A negative‐doughnut distal resection margin less than 5 mm does not affect prognosis in rectal cancer. J Surg Oncol. 2018;118(3):536-43.
28. McAnena OJ, Heald RJ, Lockhart-Mummery HE. Operative and functional results of total mesorectal excision with ultra-low anterior resection in the management of carcinoma of the lower one-third of the rectum. Surg Gynecol Obstet. 1990;170(6):517-21.
29. Morlote DM, Alexis JB. Is the routine microscopic examination of proximal and distal resection margins in colorectal cancer surgery justified?. Ann Diagn Pathol. 2016;23:35-7.
30. Rubbini M, Vettorello GF, Guerrera C, et al. A prospective study of local recurrence after resection and low stapled anastomosis in 183 patients with rectal cancer. Dis Colon Rectum. 1990;33(2):117-21.
31. Rutkowski A, Nowacki MP, Chwalinski M, et al. Acceptance of a 5‐mm distal bowel resection margin for rectal cancer: is it safe?. Colorectal Dis. 2012;14(1):71-8.
32. Skouras C, Tang E, Jamil N, et al. An evaluation of the quality of surgical resections for colorectal cancer–a district general hospital experience. Colorectal Dis. 2014;16(Suppl 2):170.
33. Terzi C, Ünek T, Sağol Ö, et al. Is rectal washout necessary in anterior resection for rectal cancer? A prospective clinical study. World J Surg. 2006;30(2):233-41.
34. Varma JS, Chan AC, Li MK, Li AK. Low anterior resection of the rectum using a double stapling technique. Br J Surg. 1990;77(8):888-90.
35. Vernava III AM, Moran M, Rothenberger DA, Wong WD. A prospective evaluation of distal margins in carcinoma of the rectum. Surg Gynecol Obstet. 1992;175(4):333-6.
36. Vilela IF, Barroso MH, Sanchez AS, et al. Utility of routine histological examination of stapler doughnuts at low anterior resection for rectal cancer. Colorectal Disease. 2018;20(Suppl 4):126.
37. Wlodarczyk J, Gaur K, Mertz K, et al. Do or doughnut: A systematic review and pooled analysis on the utility of pathological evaluation of the anastomotic doughnut in oncological colorectal operations. Colorectal Disease. 2022;24(1):8-15.
38. Bujko K, Rutkowski A, Chang GJ, Michalski W, Chmielik E, Kusnierz J. Is the 1-cm rule of distal bowel resection margin in rectal cancer based on clinical evidence? A systematic review. Ann Surg Oncol. 2012;19(3):801-8.
